# Phage-Based Artificial Niche: The Recent Progress and Future Opportunities in Stem Cell Therapy

**DOI:** 10.1155/2019/4038560

**Published:** 2019-04-03

**Authors:** Kshitiz Raj Shrestha, So Young Yoo

**Affiliations:** ^1^BIO-IT Foundry Technology Institute, Pusan National University, Busan 46241, Republic of Korea; ^2^Research Institute for Convergence of Biomedical Science and Technology, Pusan National University Yangsan Hospital, Yangsan 50612, Republic of Korea

## Abstract

Self-renewal and differentiation of stem cells can be the best option for treating intractable diseases in regenerative medicine, and they occur when these cells reside in a special microenvironment, called the “stem cell niche.” Thus, the niche is crucial for the effective performance of the stem cells in both *in vivo* and *in vitro* since the niche provides its functional cues by interacting with stem cells chemically, physically, or topologically. This review provides a perspective on the different types of artificial niches including engineered phage and how they could be used to recapitulate or manipulate stem cell niches. Phage-based artificial niche engineering as a promising therapeutic strategy for repair and regeneration of tissues is also discussed.

## 1. Introduction

Stem cells are undifferentiated cells that can self-renew and can differentiate into multiple lineages based on the provided signal, holding great promise for the repair, regeneration, and reconstruction of tissues and organs. They have very low immune rejection compared to fully differentiated cells and their multipotency to differentiate into the specific cell types [1, 2]. These merits are dependent on their surrounding microenvironment in which the stem cells reside, called “stem cell niche” [3]. Nowadays, stem cell researchers are focusing their attention on various stem cell niches. Since the implanted cells should reside in a special microenvironment to achieve desirable functions, therefore, we should consider the ways to provide a special microenvironment so as to mimic the naive stem cell microenvironment [4, 5].

In this review, we discuss about the recent progress and future opportunities in artificial stem cell niches. Firstly, we highlight the components of the stem cell niche and their function. Then, we discuss the proposed artificial niche. Lastly, we describe the engineered phage as an artificial niche and its promising application in tissue engineering.

## 2. Stem Cell Niche

Stem cells reside in the special microenvironment that consists of cellular and noncellular components that provide structural and functional cues that are various biophysical, biochemical, and mechanical cues including cell to cell contact, growth factors, and stiffness. These factors contribute to the regulating stem cell function *in vivo* [3, 5]. The research on the niche is increasing at an exponential rate as this is the governing factor for stem cell self-renewal and differentiation as well as other important biological phenomena [1, 4].

The stem cell niche is very important for the smooth performance of stem cells; determining its fate and the absence of which leads to loss of those functions. The concept of the niche was proposed around 4 decades ago but is best understood today due to the understanding of the microenvironment by using recent tools [6–8]. A stem cell, according to its niche, can undergo four different fates: (a) quiescent, (b) symmetric divisions (giving rise to two daughter stem cells), (c) asymmetric divisions (giving rise to one daughter stem cell and one differentiated cell), and (d) divisions with loss of self-renewal (giving rise to two differentiated progeny) [1].

**Figure 1:**
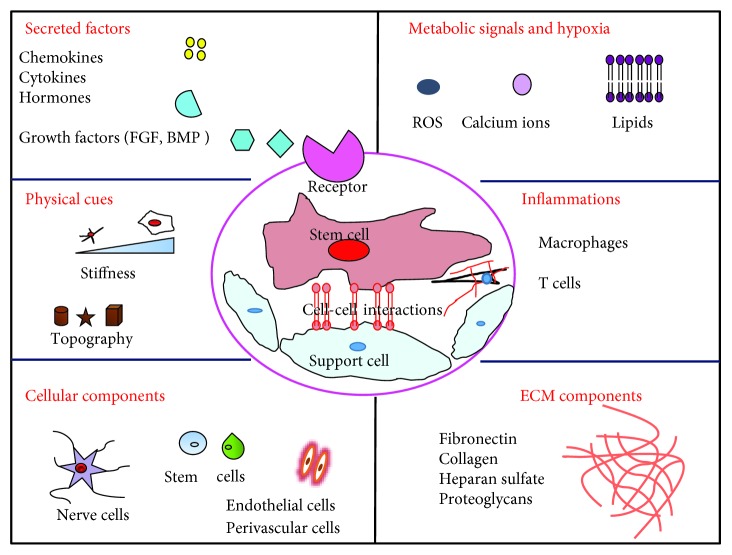
Generic components of the stem cell niche. The stem cell niche is the special microenvironments that consist of many factors such as cellular and secreted factors, ECM proteins, physical parameters, metabolic signals, and immunological factors. All the parameters function in a coordinated way to attain a specific goal. ROS: reactive oxygen species; ECM: extracellular matrix (adapted and modified from [3]).

Every stem cell niche is distinct and specific in its own way and the way they interact with the neighboring cell population. But there are common features that are shared by all the different types of stem cell niches. The generic components of the stem cell niche are illustrated in Figure 1. The advancement in scientific technology has led to a successful understanding of the stem cell niche.

### 2.1. Cellular Components in the Stem Cell Niche

The stem cell niche consists of different types of cells, and each of the cells has a specific function. For example, the hematopoietic stem cell (HSC) niche contains various cell types like osteoblasts, vascular, neural, macrophages, and immune cells, and each of them has a specific function [9–11]. Nowadays, scientists are debating on the differential functions of endosteal and perivascular niches, mainly, whether they have specialized roles or whether there is harmonized regulation of HSC, and as a result, there is an overlap of function [12]. The stem cell and the niche cells communicate with each other by either direct cell contact physical interaction or indirectly secreted factors. Heterogeneous cell-cell interactions are always present and often show complex bidirectional signaling [13, 14]. Direct contact is mediated by a range of receptors including cell-cell adhesion molecules and receptors with membrane-bound ligands. On the other hand, there is the presence of blood vessels which transport long-range signals as well as a channel for recruitment of circulating cells into the niche [3].

#### 2.1.1. Cell-Cell Adhesion Molecules

Cell adhesion molecules are membrane-associated cell surface glycoproteins involved in numerous cellular processes including cell recognition, adhesion, migration, differentiation, and cancer metastasis. They are also responsible for exchanging information from ECM to the cell [15, 16]. Based on the different structures and functions, cell adhesion molecules are classified into immunoglobulin (Ig) superfamily cell adhesion molecules (CAMs), integrins, cadherins, and selectins [17]. It has been reported that E-selectin is expressed by bone marrow endothelial cells in the vascular HSC niche, thus promoting the proliferation of HSC. The authors illustrated that HSC quiescence was improved and self-renewal potential was increased after the antagonists of E-selectin were administered. This showed E-selectin encourages HSC proliferation and is an important component of the vascular niche [18].

#### 2.1.2. Membrane-Associated Proteins

Adhesion molecules (support cells) tether the stem cells and also provide a favorable microenvironment for the biological functionality of cells; however, the underlying mechanism is not clearly understood. These types of cell-cell interaction are mainly governed by the cadherin protein family [19]. In order to fully understand the *in vivo* mechanism related to stem cells, the scientific community is mimicking the same in *in vitro*. Apart from physical cues, the biochemical cues have also been widely reported to affect stem cell fate by targeting specific signaling pathway, such as the Wnt signaling pathway in the HSC, *β*1 integrin-activated MAPK signaling, and Notch signaling in the development of the nervous system [20–22]. Notch signaling is a significant signaling pathway functioning through Notch receptors and their ligands Jagged and delta. These transmembrane proteins are expressed by stem cells and their supporting cells in different tissues. Notch signaling plays a vital role in controlling cell function during embryonic development and in adult tissues for stem cell self-renewal and differentiation [22–25].

### 2.2. Soluble Niche Effectors

Secreted and membrane-bound factors like chemokines, cytokines, hormones, growth factors (GFs), and Wnt directly bind surface receptors on the stem cells to modulate stem cell fate [26, 27]. Soluble candidate molecules, developmental morphogen proteins such as fibroblast growth factors (FGFs), bone morphogenetic proteins (BMPs), Wnt, or hedgehog proteins can be found in many niches across different species, ranging from the fruit fly to mammals. For instance, FGF, Shh, and Wnt3a have appeared as the candidates for regulating HSC self-renewal [28–30]. The soluble factors found so far are the expressing proteins during normal tissue development. In order to better understand the role of stem cells in various physiological and pathological conditions and exploit these cells for the repair and regeneration, stem cell researchers are working on the precise cell intrinsic and cell extrinsic regulators of key stem cell function [31–36].

### 2.3. Extracellular Matrix (ECM) Components

ECM is protein- and sugar-rich cross-linked gel networks that surround stem cells thus providing structure and organization as well as mechanical and biochemical signals [37]. They are the important component in the stem cell niche as they can directly or indirectly modulate the maintenance, proliferation, self-renewal, and differentiation of stem cells [37]. Many cellular phenomena including stem cell functions were powerfully governed by ECM. They can be either 2-dimensional sheets like basal lamina or 3-dimensional fibrillar polymer networks [36]. ECM directly interacts with cells via cell integrin receptors and regulates cellular activity as well as morphology by providing various kinds of instructive cues such as physical, biochemical, or mechanical cues [38–40]. Stem cells in *in vitro* conditions may not be active without these governing factors, so a proper external niche should be provided. The external niche would revolutionize the cell culture, and if this niche can be maintained *in vivo*, then it can prove to be a great boon for cell and other transplantation studies [1, 41].

### 2.4. Metabolic Signals

Apart from the above-mentioned components of the stem cell niche, there are many metabolic signals like calcium ions, reactive oxygen species (ROS), and lipids, which can influence the stem cell functions [42–44]. HSC, cardiac progenitor, and many other cell populations reside in a low oxygen tension microenvironment that contributes to their survival and maintenance. Kimura and Sadek demonstrated that cells in hypoxic conditions perform glycolysis thus expressing increased levels of hypoxia-inducible factor 1*α* (HIF1*α*) [45]. It has been reported that HSC located close to the bone's endosteal surface is exposed to high calcium ion concentration. Moreover, it expresses a high level of calcium-sensing receptor, and the lack of these receptors leads to the loss of stem cell ability to find their way back into the niche [42].

### 2.5. Immune Factors

Many cells of the innate and adaptive immune system migrate in and out of the tissue. The immune cells modulate to perform stem cell function. These cells also provide niche regulations during tissue damage and inflammations [3]. Fujisaki et al. demonstrated Treg cells accumulate in the hematopoietic stem/progenitor cell (HSPC) niche and might provide this niche with immune privilege mechanism, facilitating transplanted allo-HSPCs to escape from allogenic rejection. This mechanism of the HSPC niche will protect endogenous HSPCs from excessive inflammations thus will assist malignant cells to escape host immunity [46].

### 2.6. Physical Factors

Stem cells respond to the cues from the physical surroundings like stiffness, topography, and shear force, and these have an influence on stem cell fate [47]. Substrate stiffness has a profound influence on adhesion, migration, proliferation, and differentiation of numerous cells [48]. Engler et al. reported that stiffness of various organs and tissue varies from the lowest stiffness in the case of soft tissue like a nerve to the highest stiffness in the case of bone [49]. Furthermore, tissue stiffness is changed by the diseased state. For example, the stiffness of mammary tissue increases from 1 kPa in normal to 4 kPa during breast cancer [50]. A stem cell cultured on a standard tissue culture plate loses stemness due to the higher stiffness, so the substrate stiffness has to be modulated in order to mimic the native stem cell niche [47].

## 3. Hematopoietic Stem Cell (HSC) Niche

Out of many niches in the living system, some of the well-characterized niches are hematopoietic stem cell (HSC), muscle stem cell, neuron stem cell, and endothelial stem cell niches [41]. Here, we have discussed the HSC niche as a related example.

**Figure 2:**
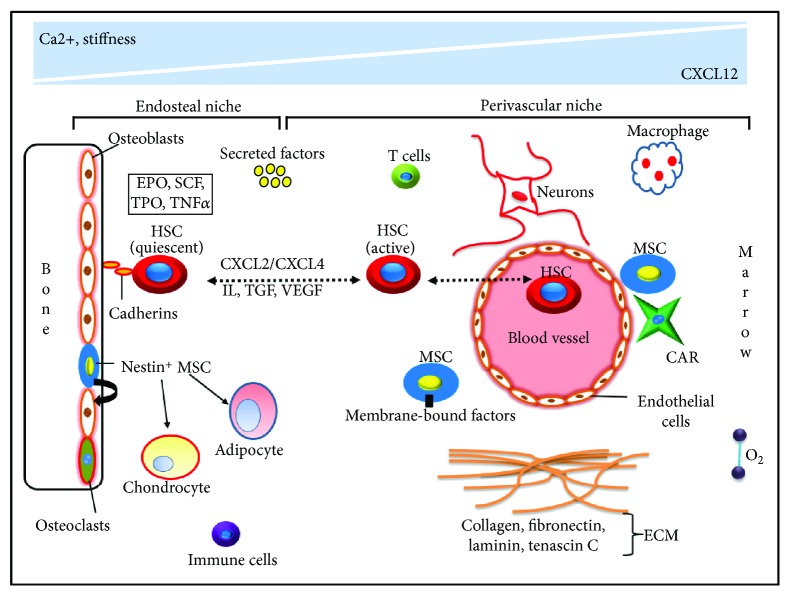
Schematic diagram illustrating the HSC niche. The HSC niche consists of endosteal and perivascular niches. There exist niche components like cellular factors (osteoblast, nestin^+^ MSC, and HSC), secreted factors (EPO, SCF, and TPO), ECM (collagen and fibronectin), metabolic signals (O_2_), immune cells (macrophages and T cells), and matrix stiffness. All the HSC niche components function in a harmonized way to attain a specific goal. CAR: CXCL12 abundant reticular cell; ECM: extracellular matrix; HSC: hematopoietic stem cell; IL: interleukin; MSC: mesenchymal stem cell; SCF: stromal cell-derived factor; TGF: transforming growth factor; TNF*α*: tumor necrosis factor alpha; TPO: thrombopoietin; VEGF: vascular endothelial growth factor. The images are not fit to scale (adapted and modified from [37, 41, 54]).

HSCs are multipotent progenitor cells with their self-renewal capacity that give rise to all the blood cells and comprise the immune system [51, 52]. They are localized in between the endosteal surface of trabecular bone close to osteoblasts and the endothelial cells that line the blood vessels. The endosteal niche (quiescent HSC) and the perivascular niche (active HSC) are two distinctive cellular entities that are present in the HSC niche [9, 53]. HSC is attached to the endosteal niche by cell-cell interactions mainly by N-cadherin (Figure 2). Next, the perivascular niche resides around small sinusoidal blood vessels related to the different stromal and neural elements. These elements regulate the differentiation of HSC and ultimately mobilization to the neural circulation.

### 3.1. Cellular Components in the HSC Niche

Different cell types like osteoblasts, vascular endothelial cells, bone marrow adipocytes, nestin-positive mesenchymal stem cells (nestin^+^ MSCs), CXCL12 abundant reticular (CAR) cells, macrophages, and neuronal cells are actively associated with the HSC niche for HSC quiescence, self-renewal, and differentiation [55–57]. It has been reported that osteoblasts influence HSC pool by regulating the stem cell number and also maintain HSC dormancy by releasing signals like the stromal cell-derived factor (SCF), thrombopoietin (TPO), and angiopoietin-1 (Ang-1) [56, 58]. Nestin^+^ MSCs are an important component of the HSC niche. Self-renewal and differentiation of MSC are regulated through vascular cell adhesion molecule-1 (VCAM-1) or via soluble factor SCF. Besides osteoblasts and MSC, endothelial cells are also important for the maintenance of HSC functional phenotype [59, 60]. These cells are important in the context of HSC mobilization, homing, and engraftment. Cytokines like fms-related tyrosine kinase 3-ligand (Flt3L), granulocyte colony-stimulating factor (G-CSF), interleukin-3 (IL-3), IL-6, IL-11, SCF, and TPO are known mediators of quiescence, self-renewal, and engraftment *in vivo* [58, 61–64]. CXCL12 is an effective chemokine expressed by HSC niche cell CAR, which is found in perivascular regions [65, 66]. CXCL2, CXCL12, G-CSF, SCF, and interleukins (IL-1/6/7/8/12) have been involved in HSC homing, migration, and retention within the bone marrow niche [54, 67].

### 3.2. Other Important Factors in the HSC Niche

The metabolic factors including calcium ions, oxygen tension, and ROS are also present in the HSC niche [68]. HSC that is located near the endosteal surface of the bone is subjected to high calcium ion. It expresses a high level of calcium receptors, and the absence of these receptors leads to the loss of stem cell ability to find their way back into the niche [42]. Bone marrow (in the HSC niche) experiences stiffness in the order of 40-50 kPa near the bone surface region whereas the central medullary region experiences stiffness in the order of ≤3 kPa [26]. HSCs inside the bone marrow, as well as the cells mobilized within the blood, also experience additional biochemical forces including hydrostatic pressure and fluid shear stress [49, 69]. Next, immune factors like Treg cells gather in the HSPC niche and might offer this niche with immune privilege mechanism, thus, facilitating transplanted allo-HSPCs to escape from allogenic rejection. This mechanism of the HSPC niche will defend endogenous HSPCs from excessive inflammations thus will assist malignant cells to escape host immunity [46].

### 3.3. Extracellular Matrix Proteins in the HSC Niche

ECM proteins like collagen IV, collagen VI, fibronectin, vitronectin, laminin, and tenascin C are widely found in the bone marrow niche. HSCs and their differentiated progeny express a variety of integrins like *α*4*β*1, *α*5*β*1, *α*L*β*2, and *α*M*β*2 [70]. The interactions between HSC and ECM are mediated by integrin, and downstream signaling pathways have been involved in HSC differentiation, quiescence, and mobilization. Near the endosteum, there are high levels of fibronectin whereas higher levels of laminin are observed in the perivascular space [53]. Collagen VI was reported as cytoadhesive substrates for different hematopoietic cell types [71]. Nakamura-Ishizu et al. showed that tenascin C is required for hematopoietic regeneration by promoting the *in vivo* and *in vitro* proliferations of hematopoietic stem and progenitor cells [72].

## 4. Cell Numbers in Stem Cell Pool

It has been reported that stem cell pool decreases with age due to the loss of self-renewal activity and terminal differentiation. Due to the various intrinsic and extrinsic factors, the pools undergo apoptosis or senescence. But, it is still unclear if what governs the stem cell choice between apoptosis and senescence [73]. Various research groups have reported the age-dependent decrease in the number of stem cell or perturbed (disturbed) cell cycle activity [74–77].

Stem cell pool size is correlated with the niche size [55]. One particular type of the stem cell may have multiple types of niche, and the nature of two niches may serve to command the state of stem cell activity [7, 78–80]. The HSC niche represents the best example of having two different niches, endosteal and vascular niches, and they function in a coordinated manner [9]. During homeostasis, the stem cell number in the niche must be kept constant by certain signals [8]. The dynamic niche can be made or damaged in response to physiological needs. Under physiological stress and pathological conditions, the demand is higher and self-renewal divisions are dominant leading to the expansion of the stem cell pool. On the other hand, symmetric differentiation division leads to a decrease in the stem cell pool in the niche compromising regeneration [1].

## 5. Creating Stem Cell Niches *In Vitro*

To overcome the issues in using single stem cells, many researchers are digging to generate an artificial platform mimicking crucial biochemical or structural aspects of the niche, “an artificial niche.” In order to imitate the natural niche in the living system, the researchers since a decade and a half have focused their study towards engineering an artificial niche so that the stem cell can be explained properly *in vitro*. Culturing cells in polystyrene plates may not mimic the *in vivo* environment as the cells are under the influence of 2-dimensional and high-stiffness cultured plates [47]. Manipulation of the culturing substrate is required so as to get the believable and reproducible results. Stiffness is generally represented by elastic modulus or Young's modulus of the materials and is represented by rigidity, flexibility, and modulus [81–84]. Tunable biomaterials alone or in combination with other technologies could assist in designing appropriate cues that are essential for an artificial niche. Biomaterials can be natural, synthetic, or semisynthetic. Biomaterials along with microfabrication platforms could be of great help in designing the artificial niche and identifying the stem cell regulators. Growth factors (GFs) and ECM are the major components for creating the artificial stem cell niche.

### 5.1. Growth Factors (GFs)

GFs are the protein molecules that have a significant role in various cellular processes ranging from cell growth, differentiation, and migration. Various GFs have been used directly or via gene therapy since many decades for the treatment of several pathological conditions, and many are being investigated for tissue engineering and regenerative medicine. GFs like FGF, PDGF, BMP2, TGF*β*1, VEGF, and IGF play important roles [85–87].

### 5.2. ECM

ECM provides “mechanical support” for neighboring cells as well as a range of “biochemical and biophysical signals” that influence the behavior of the cell. These are mainly due to the composition of ECM that comprises fibrous matrix proteins, adhesive glycoproteins, glycosaminoglycans, and proteoglycans [88]. Apart from these, ECM also acts as a modulator of intracellular signaling pathways [89].

In order to control stem cell behavior in *in vitro* condition, the material with niche-like characteristics should be selected so that it can be molecularly engineered and functionalized in order to mimic physiological condition. There exists a give and take relation at the cell/material interface. It gives the signal to the cell in the form of degradation by-products and also takes a signal from the cell by the binding and unbinding of GF from material-associated ligand [90]. Various types of materials including polymers like hydrogel and PEG and some of the natural materials like chitosan are being investigated for this purpose [91, 92]. Based on the source of origin, they can be further classified into natural and synthetic biomaterials.

#### 5.2.1. Naturally Derived Biomaterials

The biomaterials derived from the natural source and native tissues are used for modulating the stiffness by many researchers. Native tissues like collagen and glycosaminoglycan as well as natural materials like gelatin, agarose, fibrin, collagen, polyproteins, alginate hydrogels, silk hydrogels, silk-alginate hydrogels, and hyaluronic acid (HA) are widely used [93–97]. The advantages of using natural biomaterials are that they are derived from the natural source [92]. However, batch to batch variation is the major issue with the use of natural materials. Furthermore, cost, preparation/extraction time, impurity, unwanted immune reaction, and limited mechanical properties to achieve the variable elasticity are the challenging issues [92].

#### 5.2.2. Synthetic-Based Materials

To overcome the batch variations and availability of natural biomaterials, the materials of artificial origin are gaining popularity. The widely used synthetic-based materials are polyethylene glycol (PEG), polyacrylamide (PA), (meth)acrylate-based networks, poly(propylene fumarate)-co-polycaprolactone (PPF-co-PCL), poly(dimethylsiloxane) (PDMS), PEG-silica gel, polyvinyl alcohol (PVA), etc. [98–102].

Several researchers have reported the use of the above-mentioned polymers for creating the appropriate niche for cell spreading and differentiation. Substrate stiffness has been reported to be an important cue in directing MSC proliferation and differentiation [47, 49, 103]. Hydrogels alone or in their chemically modified forms are the appropriate candidates to be used for the artificial niche because the substrate is soft with a high percentage of water content. They mimic the tissues, and this can play a vital role in differentiation and other phenomena [49]. This can be a great advantage over the tissue culture plate which has a very high stiffness that does not reflect the tissue stiffness thus leading to the false result. Polyacrylamide is a popular polymer among the researchers working in creating different stiffnesses. Polyacrylamide hydrogels of various stiffnesses can be obtained by tuning the ratio of monomer (acrylamide) and cross-linker (bisacrylamide). Vertelov et al. have demonstrated that softer gels support adipogenic differentiation and stiffer gel supports osteogenic differentiation of MSC [104]. Poly(dimethylsiloxane) (PDMS) is the elastomeric material whose stiffness can be adjusted from tens of kPa to a few MPa by tuning the base to curing agent ratio [105].

## 6. Existing Artificial Niche Strategy and Challenges of Self-Renewal

Stem cells have been widely used for repair and regeneration of tissues for a long time. The hallmark of stem cells is self-renewal and differentiation, and in order to achieve this goal, stem cells are subjected to a multitude of biochemical and biophysical cues existing in their spatial locality. The differentiation of the stem cell into specific cell types has been broadly explored whereas there is limited understanding about the mechanism governing self-renewal capacity of stem cells. Self-renewal of the stem cell is the process by which stem cells divide to generate one or two daughter stem cells. It requires mechanisms that confer the capacity to divide with the maintenance of the undifferentiated state and are often multi-/pluripotent [5, 106].

The major drawbacks of the current studies with respect to modulating artificial niches are that not a single factor can fulfill and imitate the native stem cell niche. Many factors have to be taken into account to create a favorable microenvironment for the stem cell niche. Due to the technological advancement, biomaterials alone or in combination with other technologies are being used for investigation of the stem cell niche. However, they are not sufficient enough for mimicking native stem cell niches, and many factors have to be taken into considerations. Most of the work described above has illustrated that the repair and regeneration processes are mainly due to direct differentiation of stem cells or indirectly by its paracrine functions. To date, there are only a few reports of self-renewal of stem cells by exploiting the biomaterials for fabricating the biomimetic stem cell niche.

There are few literatures which use nanoscale topography [107], change in chemistry [108, 109], and substrate stiffness [110] to attain self-renewal of stem cells. It has been reported that self-renewal of MSC involves an intermediate adhesion state that suppresses differentiation and permits for long-term growth *in vitro*. MSC adipogenesis required weak adhesion supporting low intracellular protein [108, 111] while osteogenesis required large adhesions that support high intracellular protein [107, 112]. Self-renewal of MSC is favored midway between these two fates [113, 114]. Gilbert et al. showed that soft hydrogel substrates mimicking the native elasticity of the muscle (12 kPa) regulate skeletal muscle stem cell self-renewal *in vitro* and contribute to the regeneration of muscle when transplanted into mice [110]. Self-renewal of the skeletal muscle stem cell on the soft PEG hydrogel occurred even after multiple divisions. McMurray and colleagues demonstrated that nanoscale surfaces fabricated to form an array of specific depth and pitch in a square arrangement lead to the maintenance of MSC phenotype and multipotency. The authors evaluated the multipotency of MSC markers over four and eight weeks, respectively [115]. Biomaterials, signaling molecules, and cells have been used for repair and regeneration of tissues and organs in tissue. It is difficult to control the peptides that have been used on the surface of biomaterial by the chemical conjugation.

As we discussed, the HSC niche is a well-characterized niche out of many stem cell niches [41, 54, 116]. Currently, various two-dimensional and three-dimensional biomaterial platforms are being exploited to engineer the HSC niche [41, 117] However, the techniques to engineer them *in vitro* for the expansion of clinically relevant HSC population are still lacking [118]. The mutations in the hematopoiesis process can lead to pathological conditions like bone marrow failure or leukaemia. The treatment strategy in the aforementioned conditions is hematopoietic stem cell transplantation (HSCT), but there are complications in this procedure. Infections, severe graft versus host disease, and relapse contribute to mortality of patients, but the major issue remains due to low homing efficiency to the marrow cavity and failure to reengraft [119].

For mimicking the HSC niche and its application, more detailed understanding of the HSC niche along with the factors involved is needed [116]. HSC may display a variety of responses to a niche signal, and these responses will likely be magnified in multicue settings, which should be defined [120, 121]. The labelling techniques or functional assays that are currently available rarely allow in situ analysis of single, live stem cells, which may skew the characterization of stem cell responses to niche-mediated cues [122]. There are several reports discussing compositions of the stem cell niche to modulate stem cell behaviors. The researchers in this field have been trying their best to recreate the aspect of the stem cell niche to better understand the regulation of the stem cell and manipulate stem cell functions [123, 124]. However, there are still technical challenges in constructing the desired cell niches [125, 126].

To overcome the limitations mentioned above, engineered phage can be proposed as an alternative platform as it provides appropriate biophysical, biochemical, and topography cues and ECM for mimicking a native stem cell niche [127–129]. Engineered phage based on 2D films provides biophysical and biochemical cues on the proliferation and differentiation of MSC as reported [127]. Phage could induce angiogenesis and osteogenesis for MSC phage-based vascularized bone regeneration [130]. Various types of cues that are essential for mimicking a native stem cell niche are provided by the engineered phage displaying specific peptides [129, 131, 132]. Although phage does not directly help the stem cell for self-renewal or differentiation, it provides various types of physical, chemical, and topological cues that are crucial for determining stem cell fate. In addition, engineered phage induces or stimulates the native stem cell niche or modulates/controls the niche in which the stem cell resides. The engineered phage system is believed to strengthen the existing strategy that uses biomaterials and nanotopography. Engineered phage incorporated into the biomaterial provides appropriate cues for mimicking a native stem cell niche. More research is yet to be done to sort out the mechanism by which engineered phage helps to self-renewal of stem cells for repair and regeneration of damaged tissue. Herein, the multifunctional engineered phage can be considered as a better option and is discussed more in the next section.

## 7. Phage as an Artificial Niche

**Figure 3:**
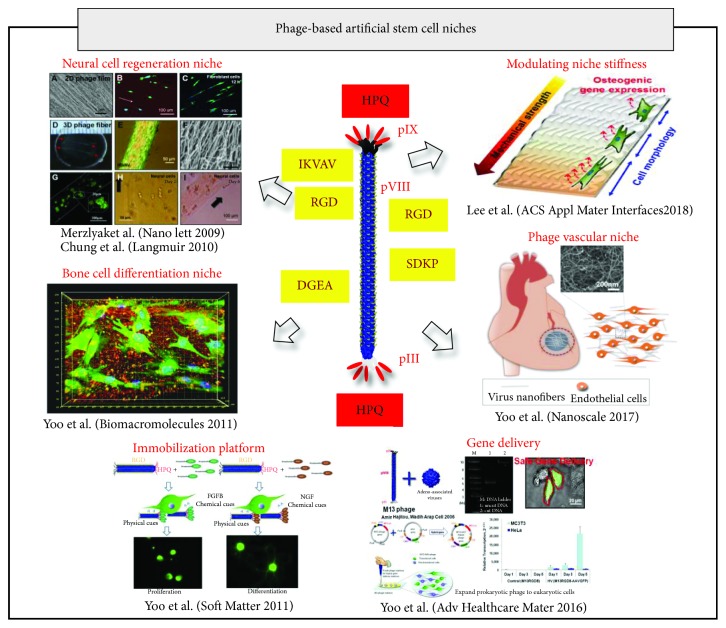
Phage engineering examples of artificial stem cell niches. Engineered nanofibrous structured phage to express short functional peptides has been utilized as tissue engineering scaffold imitating the ECM fibrous protein network for various tissue engineering purposes. The peptides displayed on the arrow are the peptide displayed on the engineered phage for providing various cues to the stem/progenitor cells to mimic a native stem cell niche. Adapted from [131, 132, 144, 146, 148, 149].

Bacteriophages (phages) are viruses that can infect bacterial host cells. They are classified based on the genetic materials, the structure of capsids containing their genome, and mechanisms of mRNA production [133]. Phage is being widely exploited in biomedical sciences and other allied areas after its discovery a century ago [134, 135]. Phage has been exploited for the detection of various antigens and effluent for a long time [136]. Several authors have reported that the presence of these peptides influences behaviors like viability, cell adhesion, proliferation, and differentiation [128, 129, 131, 137–139]. Nowadays, M13 phage is being considered as the promising tool that can be functionalized and controlled at great precision by genetic and chemical modifications of their outer protein coat with the filamentous structure of 880 nm long and 6.6 nm diameter [140]. It consists of 2700 copies of major coat protein (pVIII) that is coded by a single gene called gene VIII [141]. pVIII is generally modified for desired characteristics. One end of M13 phage is composed of five copies each of pIII and pVI while the other end is composed of five copies each of pVII and pIX [142, 143]. Moreover, the coat proteins can be genetically engineered to express short peptides so that nanofibrous structured virus expressing a functional peptide with high density has been utilized as tissue engineering scaffold imitating the ECM fibrous protein network for tissue regeneration purposes (Figure 3) [131, 132, 144–150].

Phage can be packaged in an economical manner, and also, they remain stable under different physiological stresses [151]. Phage has been reported to elicit mild immune response making it favorable for its use in a human [54]. Phage possesses the least adverse effects in the human body as they are removed from the body by lysosomal degradation [152]. The phage replication leads to accurate production of monodisperse with no error [153]. Also, several peptides can be displayed on the surface of a single phage resulting in a multifunctional nanofiber [154]. By tuning the concentration of the phage, it tends to self-assemble into different ordered structures [155]. Biomaterials are widely used for providing biochemical and physical cues for creating an artificial stem cell niche. Due to the dynamic nature of the phage, it is a very appropriate candidate for mimicking and establishing an artificial stem cell niche.

Various researchers have demonstrated that the use of engineered phage helps to provide all the different physical, mechanical, and biochemical cues thus creating a suitable artificial stem cell niche. It has been well reported that engineered phage could regulate various behaviors of cells like proliferation and differentiation [145, 147, 148]. The different types of engineered phage-based artificial cell/stem cell niches are listed in Table 1.

Merzlyak et al. genetically engineered M13 phage to display cell adhesive peptides like IKVAV and RGD on their major coat protein in a periodic and dense display. This engineered phage served as a favorable substrate providing an ECM and topographical cues for neural progenitor cell (NPC) proliferation and differentiation [132]. In the other study, to overcome the challenges of blood vessel formation in bone regeneration, Wang et al. exploited fibronectin-derived peptide RGD displayed on M13 phage and integrated with a 3D-printed MSC-seeded bioceramic scaffold to form a virus-activated matrix (VAM). Here, RGD-phage nanofibers and unique ridge/groove nanotopography served as an ECM helping osteoblastic differentiation of MSC without supplements thus leading to a successful *in vivo* regeneration of vascularized bone [130]. In both of these studies, engineered phage displaying peptides provided biochemical and topographical cues for providing a biomimetic niche so as to modulate stem cell fate. In the next study, Wang et al. fabricated M13 phage films by layer-by-layer self-assembly for induction of iPSC differentiation into osteoblast cells without any chemical supplements [129]. They showed phage-based matrices function as a substrate for generating a safe and efficient cell source apart from various cues.

Yoo et al. demonstrated an early osteogenic differentiation of mouse preosteoblasts (MC3T3) on phage engineered with DGEA peptide matrices. The groups constructed the membrane protein varying one single amino acid from DGEA or RGD in order to obtain DGDA, EGEA, or RGE phage. The response was DGEA peptide-specific showing that phage-based cues can be controlled by genetical engineering [148]. Thus, MC3T3 cultured on engineered phage matrices showed outgrown morphologies with the efficient spreading of the cells expressing early bone markers. Also, Yoo et al. reported synergistic roles of immobilized growth factors and genetically engineered phage in controlling morphology and growth of NPC [147]. They engineered M13 phage to express HPQ and RGD on their major and minor coat proteins to form nanofibrous matrices that could immobilize FGF and NGF. This engineered phage system provides biochemical, ECM, and topographical cues that mimic the native stem cell niche and is promising for tissue engineering and regenerative medicine. The genetic engineering of the phage can be an alternative to using various types of biomaterials.

Zhu et al. demonstrated that genetically engineered M13 phage with displayed functional peptides like PDPLEPRREVCE (PD-phage) and YGFGG (YG-phage) supported MSC proliferation and differentiation. The formation of the phage film with grooved structures by the layer-by-layer method can be used as a scaffold for MSC growth. They showed that phage concentration and types of functional peptides on phage nanofiber control the morphology, proliferation, and differentiation of MSC. Moreover, they concluded that engineered phage incorporated into the scaffold for governing its surface topography offers a promising model for the research related to stem cell niches [127].

Yoo et al. demonstrated that the presence of angiogenic peptide SDKP on pVIII and integrin-binding peptide RGD on pIII of M13 bacteriophage induced angiogenesis by recruiting and activating endothelial and/or stem cells and has the potential to restore tissues after ischemic injury [131]. In their study, micropatterned surfaces with engineered phage produced the highest aspect ratios and order parameters for the directional growth of human endothelial cells. Moreover, the results of the *in vitro* tube formation assay and *in vivo* Matrigel plug assay also showed that phage nanofibers provide essential topological cues to the biochemical cues of RGD and SDKP, and both the cues are critical for mimicking the stem cell niche for angiogenesis.

Our ongoing work on angiogenic differentiation showed a promising result about the use of phage (unpublished). We found that the cells were not proliferating and slowly started to die when the cells were only provided physical cues as one cue may not be sufficient in creating a special microenvironment for the cells. Interestingly, on incorporating the phage into the system, cells were adhered to the substrate and proliferated well. The engineered phage provided biochemical and topographical cues to the physical cues of the system. We propose a stem cell niche mimicking system by exploiting engineered phages.

In another study, Lee et al. demonstrated the genetically engineered M13 phage matrix modulating matrix stiffness together with providing functional peptides expressed on phage surfaces to interact with cells. This engineered phage matrix could modulate osteogenic differentiation. The authors fabricated nanofibrous RGD peptide- and HPQ peptide-engineered phages and combined them with streptavidin (for HPQ-streptavidin binding) or with PDDA (for negatively charged phage-positively charged PDDA binding) to control the stiffness of the phage matrix. This biomimetic self-assembly template assembly of engineered phage mixed with polymer helps in controlling structural and mechanical cues with different stiffnesses, thereby promoting the appropriate stiffness required for the cells to adhere and differentiate into osteogenic expressing cells [144].

In addition, the phage can also be exploited as a scaffold and vector for gene delivery. Phage was utilized as a versatile nanoink for creating 3D cell-laden scaffold printing [156]. In this study, the preosteoblast cells (MC3T3) within the scaffold showed increased proliferation and differentiation which was dependent on phage concentration. The phage-based bioink closely mimics natural structural proteins in the ECM and addresses the recent issues in bioprinting using the scaffold for fabricating the stem cell niche. Partial success had been achieved through biomimetic 3D scaffolds, the use of growth factors like vascular endothelial growth factor (VEGF), or potent cell sources such as stem cells or mature vascular cells [139]. Yoo et al. have demonstrated that the M13-adeno-associated virus phage matrix induced GFP expression into mammalian cells as a novel tissue engineering material with gene delivery functions and might possess biomedical applications including therapeutic patches [149].

Therefore, the phage can induce *in vivo* tissue repair and regeneration by mimicking and inducing the native stem cell niche. An engineered phage displaying other functional peptides could also be used for the regeneration of tissues and organs. Evaluation on the safety of a phage-based artificial niche may need to be performed more in future studies.

## 8. Conclusions

Stem cells have great potential for the regenerative medicine and treatment of various diseases. Due to the technological development and better understanding of stem cell biology, it has shed light on the importance of the stem cell niche in both the physiological and pathological conditions. Various types of biomaterial and microfabrication technologies are being employed for creating an artificial stem cell niche. In this review, we introduced different natural and synthetic biomaterials that are being used to create an artificial niche. Nowadays, the genetically engineered phage is being widely exploited as a suitable candidate for artificial niche and is a promising tool for stem cell-based therapies. Taken together, phage along with existing technology needs to be customized for mimicking a native stem cell niche for successful clinical application of stem cells.

## Figures and Tables

**Table 1:** Engineered M13 phage-based artificial cell niches.

SN	Peptide sequence expressed	Progenitor/stem cell	Influence on cell fate	References
1	PDPLEPRREVCE and YGFGG	MSC	Morphology, proliferation, and differentiation of MSC is enhanced	Zhu et al. [127]
2	RGD and PHSRN	MSC	Osteoblastic differentiation of MSC without osteogenic supplements	Wang et al. [128]
3	RGD, PHSRN, ALKRQGRTLYGFGG, and KIPKASSSVPTELSAISTLYL	iPSC	Differentiation into osteoblasts in the absence of osteogenic supplements	Wang et al. [129]
4	RGD	MSC	Vascularized osteogenesis in 3D-printed bone scaffolds	Wang et al. [130]
5	RGD and SDKP	EC	Induced angiogenesis by recruiting and activating EC; potential to restore tissues after ischemic injury	Yoo et al. [131]
6	RGD and IKVAV	NPC	Favorable substrates for NPC proliferation and differentiation	Merzlyak et al. [132]
7	RGD and HPQ	MC3T3, ASC	Stiffness platform for modulating cell expansion and differentiation	Lee et al. [144]
8	RGD and HPQ	NPC	Synergistic roles of immobilized growth factor and phage in controlling NPC morphology and growth	Yoo et al. [147]
9	DGEA	MC3T3	Early osteogenic differentiation of mouse preosteoblasts MC3T3	Yoo et al. [148]
10	RGD	NPC	Enhanced directional proliferation and differentiation of NPC	Chung et al. [150]
11	RGD and DDYP	MC3T3	Biomimetic nanoink showed enhanced proliferation and differentiation	Lee et al. [156]

EC: endothelial cells; iPSC: induced pluripotent stem cells; NPC: neural progenitor cells; MSC: mesenchymal stem cells; ASC: adipose-derived stem cells; MC3T3: preosteoblast cells.
